# A Systematic Review of the Utility of Bromocriptine in Acute Peripartum Cardiomyopathy

**DOI:** 10.7759/cureus.18248

**Published:** 2021-09-24

**Authors:** Marheb Badianyama, Prasanta K Das, Sai Rakshith Gaddameedi, Sonia Saukhla, Tejaswini Nagammagari, Vandana Bandari, Lubna Mohammed

**Affiliations:** 1 Internal Medicine, California Institute of Behavioral Neurosciences & Psychology, Fairfield, USA

**Keywords:** prolactin, pregnancy, heart failure, bromocriptine, cardiomyopathy, postpartum, peripartum

## Abstract

In formerly healthy females, acute heart failure (HF) of an unknown cause that develops during the last weeks of gestation or in the first months after childbirth is known as peripartum cardiomyopathy (PPCM). This study aimed to establish the therapeutic value of combining bromocriptine with conventional HF treatment on left ventricular ejection fraction (LVEF), death, thromboembolic events, left ventricular (LV) dysfunction recurrence in subsequent pregnancies in PPCM women, and newborn children's outcomes. We conducted a systematic review to find clinical studies that described the utility of bromocriptine in addition to conventional HF treatment compared to conventional HF treatment only in the management of acute PPCM. Four databases comprising records from July 10, 2001, to July 10, 2021, were analyzed, including PubMed (MEDLINE), Google Scholar, Scopus, and the Cochrane Library. We discovered 4,717 potentially eligible records across all the databases. According to our eligibility criteria, we included six studies consisting of 263 patients in this review. Bromocriptine combined with conventional HF therapy led to an 11.37% increase in LVEF (mean difference: 11.37; 95% confidence interval [CI]: 9.55-13.19; p-value = 0.001) after six months compared to conventional HF treatment only. Notably, bromocriptine combined with conventional HF treatment reduced mortality associated with PPCM, and no thromboembolism events were recorded in the 263 patients. PPCM is a severe condition affecting women globally. In this study, the combination of bromocriptine with conventional HF treatment enhanced the LVEF of women with acute PPCM and their clinical outcomes.

## Introduction and background

Peripartum cardiomyopathy (PPCM) is a potentially dangerous idiopathic type of cardiomyopathy that causes heart failure (HF) during the final weeks of gestation or within months after childbirth, secondary to left ventricular dysfunction defined as an ejection fraction of <45% or fractional shortening <30% with or without left ventricular dilation, in the setting of no recognizable cause of HF or earlier structural cardiac pathology [1,2]. It is crucial to consider alternative diagnoses as PPCM remains a diagnosis of exclusion. The estimated global prevalence of PPCM is about 1 in 1,000 pregnancies [3]. Factors predisposing to developing acute PPCM include older age, women of African ethnicity, multigravida, multiparity, pre-eclampsia, diabetes, malnutrition, and a low socioeconomic status [2,3]. Moreover, there are trends toward an increased incidence in some areas worldwide, including 1 in 100 live births in Nigeria, 1 in 299 live births in Haiti, 1 in 1,000 pregnancies in South Africa, and 1 in 1,149 to 1 in 3,189 pregnancies in the USA [4-6]. The current mortality rate ranges between <1% and >30%, depending on the study population, the clinical definition of PPCM, and access to quality healthcare services [3].

Although the etiology of PPCM remains unknown, a combined "two-hit" mechanism involving host susceptibility and systemic angiogenic imbalance is critical to the pathogenesis of this disease [7,8]. Factors contributing to PPCM include genetic mutations, decreased serum selenium, viral disorders, immune-mediated cytokines, autoimmunity, increased oxidative stress, inappropriate responses to hemodynamic changes during pregnancy, and upregulation of angiogenesis inhibitors [9-12]. Notably, the proteolytic degradation of the nursing hormone prolactin generates a smaller 16 KDa prolactin molecule (16 KDa PRL), also known as vasoinhibin, which prevents angiogenesis and promotes cellular apoptosis [10,13,14]. Vasoinhibin also stimulates microRNA-146a expression on endothelial cells, which subsequently drives endothelial cell injury and impairs cardiomyocyte metabolism, resulting in myocardial dysfunction [10,13]. In addition, PPCM is marked by high soluble Fms-like tyrosine kinase 1 (sFlt-1), a protein derived from the placenta, which inhibits vascular endothelial growth factor and plays an equally critical role in promoting pre-eclampsia, thus indicating the potential interaction amid these two disorders observed in previous studies [15].

The standard management guidelines of acute HF and joined obstetric care apply for women presenting with acute HF due to PPCM during pregnancy [16,17]. Furthermore, based on the pathological role of the 16 KDa prolactin fragment in this disease, treatment with bromocriptine could prevent the development of PPCM by binding dopamine D2 receptors, thereby inhibiting the pituitary release of full-length prolactin [18]. Several studies have demonstrated that bromocriptine, in combination with conventional HF therapy, appears to benefit left ventricular ejection fraction (LVEF) and maternal morbidity and mortality in women with acute PPCM [19,20]. However, the therapeutic value of bromocriptine combined with conventional HF treatment has not been firmly established chiefly due to the limited sample size included in previous studies, which have yielded results that cannot be considered definitive.

For the abovementioned reasons, we systematically searched through the available literature to ascertain the therapeutic value of bromocriptine combined with standard HF treatment in women with an acute PPCM diagnosis and its impact on the growth of their newborn children.

## Review

Methods

We implemented the Preferred Reporting Items for Systematic Reviews and Meta-Analyses (PRISMA) 2020 guidelines to design and describe the findings of this systematic literature review [21].

Search Strategy

We thoroughly searched through PubMed (MEDLINE), Cochrane Library, Google Scholar, and Scopus. We used appropriate keywords and Medical Subject Heading (MeSH) terms to identify all potentially relevant articles describing the impact of bromocriptine combined with standard HF treatment on the cardiovascular outcomes of women diagnosed with new-onset PPCM. The keywords used included peripartum, postpartum, cardiomyopathy, bromocriptine, heart failure, pregnancy, and prolactin. We applied the Boolean method to combine the keywords and MeSH terms to synthesize a uniform search through the various databases. We thoroughly checked all our records after excluding duplicates to avoid missing potentially relevant articles. We subsequently studied the retrieved records' titles and abstracts and further assessed them for relevance.

Inclusion and Exclusion Criteria

We restricted our search to online records issued in English, available as free full texts, including human participants, and issued from July 10, 2001, to July 10, 2021. We selected studies for inclusion according to predetermined criteria, which included female sex, reproductive age, all races, a clinical diagnosis of acute PPCM, comparison of outcomes between standard HF therapy with bromocriptine, and standard HF therapy only during a follow-up interval of at least six months. We also included studies in which the primary or secondary endpoint was left ventricular function recovery (i.e., LVEF ≥ 50%) or improvement (i.e. increased LVEF ≥ 10 absolute percent unit or New York Heart Association [NYHA] functional class improvement of dyspnea by one class) from baseline and the clinical outcomes listed in Table 1. We restricted our choice of studies to meta-analyses, systematic reviews, clinical trials, and observational prospective and retrospective cohorts. We used the population, intervention, comparison, and outcomes (PICO) criteria as the primary framework for our eligibility criteria.

**Table 1:** Study inclusion criteria. Key: P, population; I, intervention; C, comparison; O, outcomes; LVEF, left ventricular ejection fraction; NYHA, New York Heart Association.

P	Sex	Females
	Age	Reproductive age
	Race	All
	Peripartum cardiomyopathy clinical definition	(1) Heart failure occurring between the final four weeks of gestation and up to five months following childbirth, (2) heart failure of an unknown cause, (3) absence of structural cardiac pathology before the final four weeks of gestation, and (4) echocardiography showing impaired left ventricular systolic function, specified as LVEF <45% or fractional shortening <30% or left ventricular end-diastolic diameter >2.7 cm/m^2^.
I	Intervention	Bromocriptine and conventional heart failure treatment
C	Comparison	Conventional heart failure treatment
O	Clinical outcomes	Maternal indices: Cardiac death, all-cause mortality, NYHA heart failure functional classification, recurrent left ventricular dysfunction in a subsequent pregnancy, and evidence of thromboembolism (i.e. myocardial infarction, venous thromboembolism, and cerebrovascular accident). Newborn children indices: growth and death.

We excluded articles denoting the management of other etiologies of HF and treatment with bromocriptine for other medical conditions. We further excluded all other types of research study designs and reports. Table 1 lists our study's inclusion criteria using the PICO framework.

Data Selection and Extraction

Two researchers (MB and PKD) independently selected and extracted the relevant studies. The two researchers resolved disagreements over eligibility by discussing the study design, intervention implemented, outcomes measured, and the relevance to our inclusion and exclusion criteria. We solicited a third reviewer (SRG) when we could not reach a uniform consensus.

We identified 4,717 potentially eligible records across all the databases. Based on our eligibility criteria, six reports consisting of 263 patients form the basis of this review. The reports included two randomized controlled trials, three systematic reviews of which two were also meta-analyses, and one prospective cohort study that evaluated bromocriptine's utility in combination with standard HF treatment in the management of severe new-onset PPCM.

From the studies included, we retrieved the following data: (i) surname of the principal author and the publication date, (ii) study overview (i.e., study site, design, sample size, enrollment timeline, and duration of follow-up), and (iii) general features of the study population (mean age, LVEF, the severity of dyspnea based on the NYHA functional classification of HF, and the study findings reported using relative risk [RR] and odds ratio [OR] accompanied by 95% confidence intervals [CI], and the respective p-values).

Study Quality Appraisal

We assessed each study for the potential risk of bias. We evaluated clinical trials using the revised Cochrane risk of bias 2 (RoB 2) tool. The Newcastle-Ottawa Scale (NOS) for assessing the quality was implemented to evaluate cohort studies. We assessed systematic reviews and meta-analyses using the Assessment of Multiple Systematic Reviews 2 (AMSTAR 2) tool. Using the latest Cochrane RoB 2 tool, each randomized controlled trial was scrutinized for the presence of potential biases based on five risks of bias. Each risk of bias was scored as either low, high, or moderate. Subsequently, the overall risk of bias was also reported as evoking low, high, or moderate risk. Table 2 shows the results of the revised Cochrane RoB 2 tool.

**Table 2:** Assessment of clinical trials using the revised Cochrane risk of bias 2 tool. Key: RoB, risk of bias; LR, low risk; MR, moderate risk; HR, high risk.

First author (year)	Random allocation	intervention non-adherence	Incomplete results	Inadequate assessment of the outcomes	Selective reporting	Final RoB judgment
Sliwa et al. (2010) [22]	MR	LR	LR	LR	LR	LR
Yaméogo et al. (2017) [23]	LR	LR	LR	LR	LR	LR

Similarly, cohort studies were subjected to quality appraisal via the NOS. We assessed and scored the studies based on the selection process of the study population enrolled, comparison, and results reported through this tool. We interpreted the final scores as either good, fair, or poor quality. Table 3 shows a summary of the NOS of cohort studies included.

**Table 3:** Summary of the Newcastle-Ottawa Scale for cohort studies. Key: 1, the exposed group represents general population; 2, the non-exposed group is selected from the same population as the exposed group; 3, appropriate documentation of outcome; 4, absence of study outcome at the beginning of the cohort; 5, controlled study characteristics allow for comparison of the exposed group to non-exposed group; 6, blinded assessment of results; 7, adequate length of follow-up period; 8, complete or nearly complete follow-up of participants; NOS, Newcastle-Ottawa Scale; AHRQ, Agency for Healthcare Research and Quality; Y, yes; PY, partial yes.

First author (year)	Selected population (/4) 1 2 3 4	Comparison (/2) 5	Results (/3) 6 7 8	Final NOS (/9)	AHRQ standards
Haghikia et al. (2013) [24]	Y Y Y Y	PY	Y Y Y	8	Good quality

Finally, we used the AMSTAR 2 tool to assess systematic reviews and meta-analyses based on 16 questions. We appraised the comprehensive study quality as either critically low, low, moderate, or high. Table 4 shows the results of the AMSTAR 2 tool.

**Table 4:** Summary of the Assessment of Multiple Systematic Reviews 2 (AMSTAR 2) tool. Key: RoB, risk of bias; Y, yes; PY, probably yes; N, no; NA, non-applicable.

First author (Year)	(1) PICO framework included	(2) Pre-defined methods and research proposal	(3) Design of study outlined	(4) Thorough literature search	(5) Selection of studies by two individuals	(6) Extraction of data by two individuals	(7) Record and reasons of reports excluded	(8) Detailed description of included studies	(9) Adequate RoB procedure followed	(10) Disclosure of funding sources	(11) Appropriate statistical analysis	(12) Effect of RoB of primary studies on meta-analysis result	(13) RoB considered in primary studies	(14) Investigation of heterogeneity	(15) Small study bias	(16) Potential conflicts reported	Total score (/16)	Final quality appraisal of the review
Carlin et al. (2010) [25]	Y	Y	Y	Y	Y	Y	NA	Y	Y	Y	NA	NA	Y	NA	Y	N	15	High
Saint Croix et al. (2018) [14]	Y	Y	Y	Y	Y	Y	PY	Y	Y	N	Y	Y	Y	Y	Y	N	13	Moderate
Villanueva et al. (2020) [26]	Y	Y	Y	Y	Y	Y	PY	Y	Y	Y	Y	Y	Y	Y	Y	Y	15	High

Results

**Figure 1:**
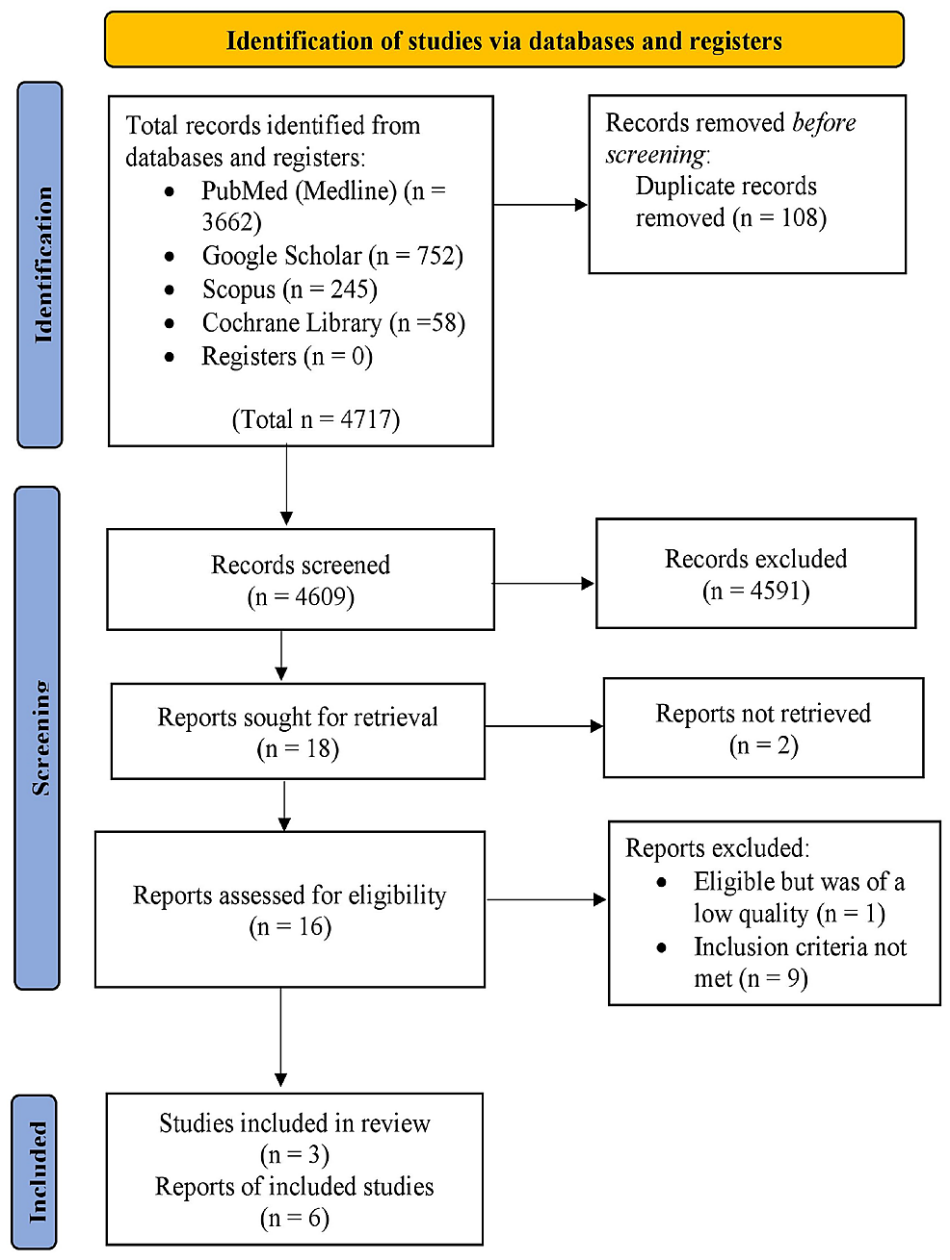
Preferred Reporting Items for Systematic Reviews and Meta-Analyses (PRISMA) flow diagram.

We identified a total of 4,717 records using the various search strategies across the databases. Out of 4,717 records, 3,662 originated from PubMed (MEDLINE), 752 from Google Scholar, 245 from Scopus, and 58 from the Cochrane Library. No other resource was used. We removed 108 duplicates manually before screening articles. The remaining 4,609 records were thoroughly screened for relevance based on titles and abstracts, after which 4,591 records were excluded due to their irrelevance to the topic, research objectives, inclusion, and exclusion criteria. Hence, 18 articles were sought for retrieval, and after checking for free full texts, we further removed two reports. We assessed 16 reports for eligibility, and nine articles did not fulfill the inclusion and exclusion criteria and were thus excluded. One article met the study's criteria but was of low quality when assessed for quality appraisal. Therefore, we included six reports in this review based on three original studies as per our eligibility criteria. These included three systematic reviews, of which two were meta-analyses, two randomized controlled trials, and one prospective cohort study. The complete PRISMA flow diagram of our review is shown in Figure 1. Tables 5-7 outline the findings of our study.

**Table 5:** Details of the studies comprised in the review. Key: Br(+), the group receiving bromocriptine with conventional heart failure treatment; Br(-), the group receiving conventional heart failure treatment only; F/u, follow-up; LVEF, left ventricular ejection fraction; MA, meta-analysis; NYHA, New York Heart Association; Prosp., prospective cohort; RCT, randomized controlled trial; SR, systematic review.

Author (year)	Country	Study design	Size	Mean age (year)	Race	Baseline LVEF (%)	Baseline NYHA class	F/u (month)
Sliwa et al. (2010) [22]	South Africa	RCT open-label	20	26 ± 8	Black	10 Br(+): 22.7 ± 8.1, p = 0.87 vs. 10 Br(-): 26.9 ± 7.6, p = 0.87	10 Br(+): II; 10 Br(-): III-IV	6
Carlin et al. (2010) [25]	Online search	SR	20	26 ± 8	Black	10 Br(+) vs. 10 Br(-) patients mean LVEF: 27 ± 7.9	II, III, IV	6
Haghikia et al. (2013) [24]	Germany	Prosp.	115	34 ± 6	White	64 Br(+) vs. 32 Br(-) patients mean LVEF: 27 ± 9	II, III, IV	6 ± 3
Yaméogo et al. (2017) [23]	Burkina Faso	RCT	96	31 ± 5	Black	48 Br(+): 37.2 ± 6.6 vs. 48 Br(-): 37.5 ± 4.8	Br(+): 14 = III, 34 = IV; Br(-): 13 = III, 35 = IV	1, 3, 6, 12
Saint Croix et al. (2018) [14]	Online search	SR and MA of RCT and Prosp.	263		Black and White	162 Br(+) and 101 Br(-) combined from Sliwa et al. [22], Haghikia et al. [24], and Yaméogo et al. [23]	II, III, IV	6
Villanueva et al. (2020) [26]	Online search	SR and MA of RCT	116	27.7 ± 5.7	Black	Sliwa, et al. [22] mean LVEF: 27 ± 7.9; Yaméogo et al. [23] mean LVEF: 36.4 ± 5.5	III, IV	6

**Table 6:** Management and outcomes measured of studies incorporated in the systematic review. Key: ACE, angiotensin-converting enzyme; ARB, angiotensin II receptor blocker; LVEF, left ventricular ejection fraction; NYHA FC, New York Heart Association Functional Class; LV, left ventricular; EF, ejection fraction.

Author (year)	Bromocriptine regimen	Conventional heart failure treatment regimen	Prophylactic anticoagulant	Outcomes measured
Sliwa et al. (2010) [22]	2.5 mg twice daily for two weeks and 2.5 mg daily for four weeks	Carvedilol 6.25-12.5 mg twice daily; enalapril 5-10 mg daily; Aldactone 12.5-50 mg daily; furosemide 80-120 mg daily	Warfarin: EF <25% or LV thrombus	Cardiovascular mortality, LVEF, worsening NYHA FC III/IV, and progress of newborn children over the six month follow-up period
Carlin et al. (2010) [25]	Same as Sliwa et al. [22]	Same as Sliwa et al. [22]	Same as Sliwa et al. [22]	Maternal cardiovascular mortality
Haghikia et al. (2013) [24]	2.5-5 mg daily, for ≥four weeks	Beta-blocker, ACE-inhibitor, ARB, mineralocorticoid receptor antagonist, diuretics	Not documented	NYHA FC after treatment, LVEF improvement (i.e. LVEF increased by ≥10% or NYHA improved by one class); LVEF recovery (i.e. LVEF ≥55%); failure to recover, and death
Yaméogo et al. (2017) [23]	2.5 mg twice daily for four weeks	Captopril 6.25-25 mg daily; furosemide 80 mg daily	Fluindione: six months for EF <25% or LV thrombus	Cardiovascular mortality, LVEF on echocardiography, and NYHA FC
Saint Croix et al. (2018) [14]	The main results were extracted from Sliwa et al. [22], Haghikia, et al. [24], and Yaméogo et al. [23]	The main results were extracted from Sliwa et al. [22], Haghikia et al. [24], and Yaméogo et al. [23]	The main results were extracted from Sliwa et al. [22], Haghikia et al. [24], and Yaméogo et al. [23]	LVEF improvement and NYHA FC
Villanueva et al. (2020) [26]	The main results were extracted from Sliwa et al. [22] and Yaméogo et al. [23]	The main results were extracted from Sliwa et al. [22] and Yaméogo et al. [23]	The main results were extracted from Sliwa et al. [22] and Yaméogo et al. [23]	Cardiovascular mortality, improvement of LVEF, and change in NYHA FC

**Table 7:** Results of the outcomes measured in each study incorporated in the review. Key: Br(+), bromocriptine with conventional heart failure treatment group; Br(-), conventional heart failure treatment only group; LVAD, left ventricular assist device; LVEF, left ventricular ejection fraction; NYHA, New York Heart Association.

Author (year)	Mean LVEF (%) after treatment including (n) % with LVEF improvement	NYHA functional class in patients alive after treatment	Thromboembolism (n) %	Deaths	Lost to follow-up	Advanced heart failure therapies (n)
Sliwa et al. (2010) [22]	Br(+): 58 ± 11; Br(-): 36 ± 11, p = 0.0007	Br(+): 9 class I; Br(-): 3 class II & 3 class III	0	Br(+): 1; Br(-): 4	0	0
Carlin et al. (2010) [25]	Not documented	Not reported	0	Br(+): 1; Br(-): 4; relative risk (RR) of death 0.25, 95% confidence interval (CI) 0.03-1.86	0	0
Haghikia et al. (2013) [24]	Br (+): 92% improved. Br(-): 72% improved. Post-therapy mean LVEF in both groups: 47 ± 19. Both groups: LVEF improved in 85%, 47% displayed full recovery, and 15% did not recover	I-II	Not reported	2	19	1 LVAD; 7 heart transplants
Yaméogo et al. (2017) [23]	Mean LVEF at six months - Br(+): 49.9 ± 2.1; Br(-): 40.9 ± 5.9, p = 0.001. Mean LVEF at 12 months - Br(+): 53.9 ± 4.1; Br(-): 45.9 ± 5.9, p = 0.001	Not reported	0	At six months: Br(+): 8 (16.6%); Br(-): 14 (29.1%), p = 0.0001. At 12 months: 0	0	0
Saint Croix et al. (2018) [14]	In Br(+) patients, LVEF improved by 11.37% at six months (mean difference 11.37, 95% confidence interval [CI]: 9.55-13.19, p = 0.001)	Overall effect not reported	0	Overall effect not reported.]	19	1 LVAD; 7 heart transplants
Villanueva et al. (2020) [26]	In Br(+) patients, LVEF improved by 15.14% at six months (mean difference 15.14, 95% confidence interval [CI]: 6.53-23.75, p = 0.0006)	Overall effect not reported	0	Br(+): 16%, Br(-): 31% (RR 0.51, 95% confidence interval [CI]: 0.25-1.06, p = 0.07)	0	0

Discussion

This section discusses the molecular pathophysiology of PPCM, diagnostic criteria, current standard therapy, and previous studies showing the role of bromocriptine use in PPCM on left ventricular (LV) function, mortality, thromboembolic events, LV dysfunction recurrence in subsequent pregnancies, and outcomes of newborn children.

The Molecular Pathophysiology of PPCM

Prolactin: Signal transducer and activator of transcription 3 (*STAT3*) activation increases the production of manganese superoxide dismutase (MnSOD), a potent inhibitor of reactive oxygen species (ROS) in the myocardium, which prevents oxidative stress while promoting the production of new blood vessels in cardiomyocytes [27,28]. Hilfiker-Kleiner et al.'s genetic mouse model showed that the knock-down of *STAT3 *gene in female mice subsequently led to the development of PPCM [13]. Notably, female mice lacking *STAT3* demonstrated high levels of the cardiac cathepsin-D enzyme, which breaks the full-length 23 KDa prolactin hormone into vasoinhibin, the shorter 16 KDa prolactin sub-fragment responsible for impaired angiogenesis and increased apoptosis of myocardial endothelial cells present in PPCM [13]. Interestingly, samples of myocardial tissues and sera from women diagnosed with PPCM showed reduced amounts of *STAT3* and, consequently, increased amounts of cathepsin-D and vasoinhibin compared to their healthy female counterparts, thus extrapolating the results observed in female mice to humans with PPCM [13].

Furthermore, the study found that treatment consisting of bromocriptine, which inhibits prolactin release from the pituitary leading to the suppression of lactation, prevented PPCM from manifesting in mice lacking *STAT3* and enhanced the cardiac output function in women with PPCM [13]. The study also revealed that vasoinhibin stimulates the expression of microRNA-146a on endothelial cells, which promotes apoptosis and impairs metabolism in cardiomyocytes [13]. Previous studies of women with PPCM have consistently demonstrated high serum levels of microRNA-146a in these patients, in contrast with patients diagnosed with familial dilated cardiomyopathy (DCMO), implying that although PPCM and DCMO may have similar genetic variants [29], the two diseases manifest through different pathophysiological pathways [13,30].

Anti-angiogenic factors: Patten et al. showed that pregnant mice deficient in peroxisome proliferator-activated receptor gamma coactivator 1-alpha (*PGC-1α*), another pro-angiogenic gene, developed severe PPCM [7]. *PGC-1α* is a prominent regulator of angiogenesis through the activity of vascular endothelial growth factor (VEGF). The study showed that pregnant mice lacking *PGC-1α* that developed PPCM were only partially salvaged by injections with VEGF, whereas the addition of bromocriptine to VEGF therapy achieved total recovery; thus, suggesting the implication of these two pathways in the pathogenesis of PPCM [7].

In late gestation, the human placenta secretes a VEGF-inhibiting protein known as soluble Fms-like tyrosine kinase 1 (sFlt-1), which halts angiogenesis, and is also markedly elevated in pre-eclamptic women [31]. In their investigation, Patten et al. further found that pregnant mice lacking *PGC-1α* developed severe HF after receiving sFlt-1 injections, indicating that sFlt-1 promotes an anti-angiogenic milieu that can trigger PPCM in susceptible mice [7]. In healthy women, placenta-derived sFlt-1 usually normalizes within 48-72 hours after delivery; however, in PPCM, sFlt-1 levels remain significantly elevated postpartum [7,31]. A cohort of women with PPCM revealed that circulating levels of sFlt-1 were more than 10 times higher four to six weeks after delivery than in healthy women; thus, supporting the pathological role of sFlt-1 in humans with PPCM [7,31]. Although the mechanism remains unclear, extra-placental sources of sFlt-1 such as peripheral blood mononuclear cells have shown to maintain high circulating sFlt-1 levels beyond 48 hours after delivery, which contribute to this disease's pathogenesis [32].

Similarly, sFlt-1 is crucial to the pathogenesis of pre-eclampsia. In a meta-analysis on the association between PPCM and pre-eclampsia, Bello et al. revealed that the presence of pre-eclampsia in women with PPCM was at least fourfold (22%, 95% confidence interval [CI]: 16-28; p-value < 0.001) than in the general peripartum population (3-5% on average) [31].

**Figure 2:**
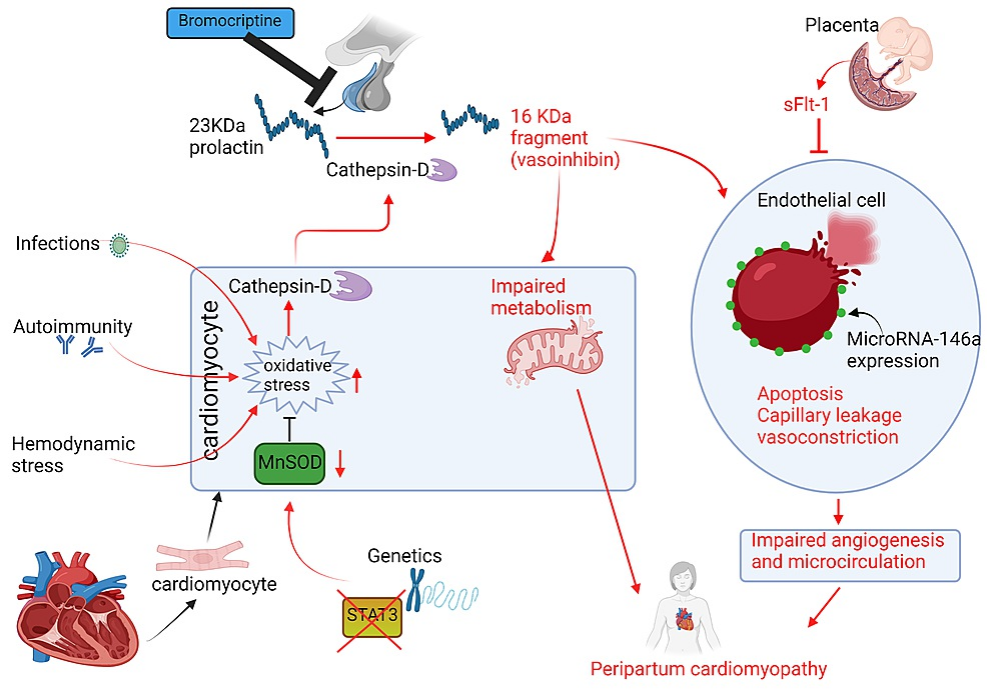
The molecular pathophysiology of peripartum cardiomyopathy (PPCM). Triggers of increased myocardial oxidative stress in the peripartum period generate the anti-angiogenic 16 KDa prolactin fragment (vasoinhibin) from the 23 KDa nursing hormone prolactin, which drives PPCM [13]. Accordingly, bromocriptine inhibits pituitary prolactin release, decreasing circulating prolactin levels and preventing PPCM. Created with BioRender.com. sFlt-1, soluble Fms-like tyrosine kinase 1; MnSOD, manganese superoxide dismutase; STAT3, signal transducer and activator of transcription 3​​​

Overall, PPCM develops through a "two-hit mechanism" [7,13,15,33]. The first hit involves the systemic anti-angiogenic hormonal environment in late gestation, characterized by high circulating vasoinhibin and sFlt-1. The subsequent trigger involves a multifactorial susceptibility hit to this anti-angiogenic environment often precipitated by genetic variations in titin (*TTN*) and troponin C1, slow skeletal and cardiac type (*TNNC1*) genes, which encode the muscle proteins titin and troponin C, respectively, viral myocarditis, autoimmunity, nutritional deficiencies, hypertension, and an abnormal response to the hyperosmolar stress during pregnancy [29,33,34]. However, additional research is required to confirm this hypothesis [15]. Figure 2 summarizes the molecular pathophysiology of PPCM.

Diagnosis of PPCM

PPCM is diagnosed when these criteria are observed [15]: HF occurring at the end of pregnancy or within the months after delivery (most commonly in the month after childbirth), a lack of a demonstrable cause of HF, and echocardiography showing LV systolic dysfunction, which is defined by LVEF <45%.

The clinical diagnosis is supported by serum cardiac protein assays, echocardiography, cardiac magnetic resonance imaging (MRI), and in some cases, endomyocardial biopsy [2].

Treatment of Acute PPCM

The disease-specific management of acute PPCM continues to pose a challenge despite the recent progress in understanding its pathogenesis. Women presenting with acute PPCM require coordinated cardiovascular and obstetric management to optimize maternal and fetal well-being and safety during pregnancy, as some standard HF drugs are contraindicated. The management guidelines for HF applied to treat HF in the general population can be implemented to treat women presenting with PPCM-induced HF after delivery [16]. In addition, practical guidance has been outlined for managing acute PPCM after childbirth, which consists of [35] (i) decongestive therapy via vasorelaxants, water pills, and non-invasive ventilatory support to reduce pulmonary edema and end-organ dysfunction; (ii) oral HF drugs (e.g. beta-receptor blockers, angiotensin-converting enzyme [ACE]-inhibitors, and mineralocorticoid receptor antagonists) to achieve better cardiac pumping function and cardiovascular outcomes; and (iii) complex HF treatment modalities including LV assist device and heart transplant when indicated.

Although bromocriptine reduces lactation, Hilfiker-Kleiner et al. have suggested that bromocriptine-induced ablactation offers the opportunity to administer oral HF therapies without harming newborn children [18]. Indeed, due to the newborn's safety concerns, several HF drugs are contraindicated during breastfeeding (e.g. sacubitril/valsartan and mineralocorticoid receptor antagonists). Most importantly, bromocriptine should be administered with anticoagulants such as heparin at a prophylactic dose to decrease the risk of thromboembolism. In addition, multiple studies have recommended combining bromocriptine with standard HF therapy as a disease-specific therapeutic agent for severe cases of PPCM [18]. This recommendation follows the low rates of thromboembolic events (i.e., myocardial infarction, cerebrovascular accident, deep venous thrombosis, and pulmonary embolism) suspected to be associated with bromocriptine in some studies [3].

**Figure 3:**
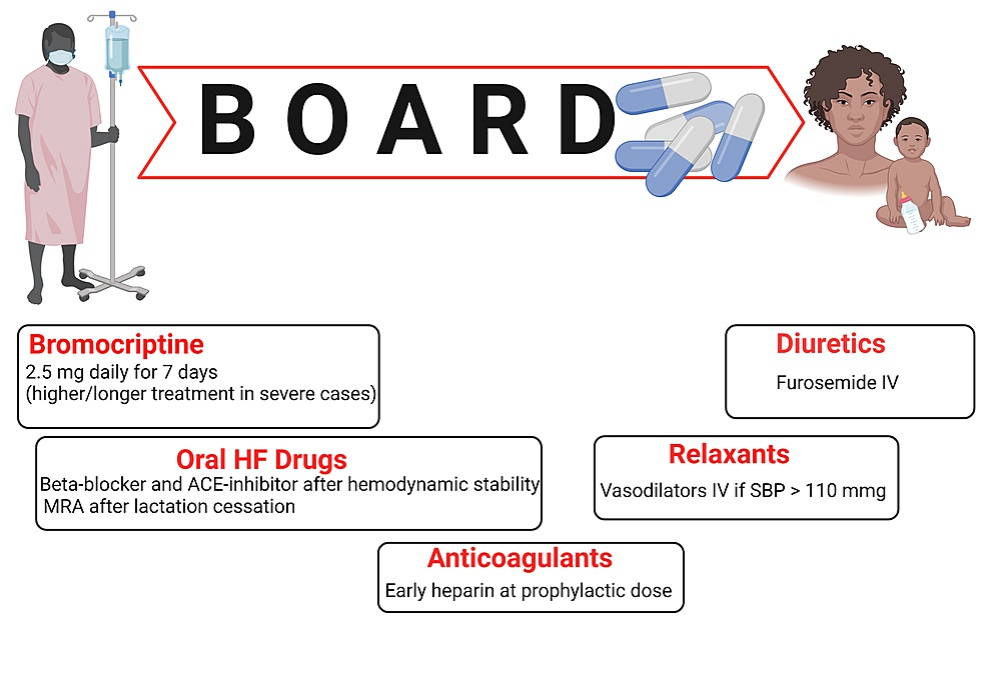
Treatment of acute peripartum cardiomyopathy (PPCM). The BOARD regimen contains bromocriptine, oral heart failure drugs, anticoagulants, relaxants, and diuretics [36]. Key: ACE, angiotensin-converting enzyme; HF, heart failure; IV, intravenous; MRA, mineralocorticoid receptor antagonist; SBP, systolic blood pressure. Created with BioRender.com.

Therefore, the primary treatments for acute PPCM are classified using the BOARD acronym, which consists of bromocriptine, oral HF drugs, anticoagulants, vasorelaxants, and diuretics [36]. Although the ideal bromocriptine regimen has not been established yet, the BOARD regimen was successfully tested in a German study involving Caucasian women with PPCM [18]. However, further research is required to test whether this regimen would benefit women with acute PPCM in other regions worldwide. Figure 3 summarizes the proposed BOARD treatment regimen for acute PPCM.

Studies on the Use of Bromocriptine in PPCM

Left ventricular function recovery or improvement and NYHA HF classification: Saint Croix et al.'s study reported that bromocriptine combined with regular HF treatment leads to an 11.37% increase in LVEF (mean difference 11.37, 95% confidence interval [CI]: 9.55-13.19; p = 0.001) after six months when compared with conventional HF treatment only [14]. Notably, Haghikia et al.'s prospective cohort study revealed that women presenting with acute PPCM and severely reduced baseline LVEF ≤25% had poorer outcomes at six months compared to patients with baseline LVEF >25%, irrespective of whether bromocriptine was introduced to the conventional HF regimen [24]. On admission, a severely reduced LVEF might be an independent prognostic factor of adverse cardiovascular outcomes at six months in women presenting with PPCM. This study was the only study to document the use of mineralocorticoid receptor antagonists in postpartum women, and no safety concerns were raised regarding the newborn children in view that mothers receiving bromocriptine could not nurse their newborn children [24]. Subsequently, the follow-up loss amounted to 19 patients because some did not agree to continue participating in the study. However, the onset of PPCM was not clearly defined in Haghikia et al.'s study [24], making it challenging to correlate disease progression and serum prolactin sub-fragment levels, which is essential to the pathophysiology of PPCM and the severity of LV dysfunction.

This pathophysiological correlation appeared more evident in Sliwa et al.'s pilot study, which recruited 20 consecutive cases of PPCM-induced acute HF with LVEF ≤35% diagnosed between the last four weeks of gestation and one month after childbirth [22]. The time of the PPCM diagnosis in this study might correlate more with increased serum prolactin sub-fragments and, therefore, the higher LV function recovery reported in the group treated with bromocriptine compared with the group receiving standard HF therapy only might indeed have been due to the use of bromocriptine. In this study [22], women receiving bromocriptine and standard HF therapy displayed significantly greater LVEF recovery (27%-58%, p = 0.012) than women receiving standard HF therapy only (27%-36%) at six months. Moreover, women on bromocriptine improved to NYHA functional class I after six months of treatment compared to those receiving standard HF therapy only, which improved to NYHA functional class II or III after six months. Furthermore, the composite endpoint of poor outcome consisting of severely reduced LVEF <35%, NYHA functional class III/IV, or cardiovascular mortality after six months was lower in study participants treated with bromocriptine combined with standard HF treatment compared to study participants treated with standard HF therapy only [22]. However, this study was small, and thus, its findings are in no way definitive.

Yaméogo et al.'s study [23], which enrolled 96 patients presenting with acute HF secondary to PPCM, reported an increase in LVEF from 37.2% on admission to 49.9% after six months and 53.9% after 12 months in patients receiving bromocriptine and conventional HF therapy while the LVEF improved from 37.5% at baseline to 40.9% after six months and 45.9% after 12 months in patients receiving conventional HF therapy only. Most importantly, the study revealed that bromocriptine combined with standard HF therapy leads to early and almost total restoration of ventricular function and reduces death related to PPCM.

Mortality: Yaméogo et al.'s study [23] reported that the six-month death rate was substantially lower in women receiving bromocriptine in addition to conventional HF therapy (16.6%, p = 0.0001) than in patients receiving conventional HF therapy only (29.1%). However, Villanueva et al.'s study showed no distinction in the risk of cardiovascular death among women receiving bromocriptine combined with conventional HF treatment and those receiving conventional HF treatment only (16% vs. 31%, respectively; relative risk [RR]: 0.51, 95% confidence interval [CI]: 0.25-1.06, p-value = 0.07) after six months [26]. Of 263 patients, nine patients receiving bromocriptine died after six months compared to 18 patients receiving standard HF treatment only. None of the nine patients receiving bromocriptine died due to side effects of bromocriptine but rather due to complications of the disease.

Thromboembolic events: More importantly, the studies included in this review noted no thromboembolic events, including cerebrovascular accidents, myocardial infarction, and venous thromboembolism [14,22-26]. The lack of this adverse finding is crucial as some studies have suggested that bromocriptine increases the risk of thromboembolism [37-40]. Except for Haghikia et al. [24], the remaining studies reported using some form of prophylactic anticoagulation in patients presenting with severe PPCM and LVEF ≤25% [14,22,23,25,26]. The administration of prophylactic anticoagulation may have played a role in the lack of thromboembolic events observed in all the studies, which further highlights the importance of anticoagulation, specifically in women receiving bromocriptine for severe PPCM who are at high risk of such adverse events.

Prognosis and left ventricular dysfunction recurrence in subsequent pregnancies: No study reported left ventricular dysfunction recurrence development in the subsequent pregnancies of PPCM women treated with bromocriptine combined with regular HF treatment compared to those treated with regular HF treatment only [14,22-26]. The lack of reporting may be attributed to the short follow-up period of the individual studies, which was limited to six months after delivery in most studies and 12 months after delivery in one study.

Outcomes of children born to PPCM mothers treated with bromocriptine: No other study except Sliwa et al.'s pilot study reported the clinical outcomes of the newborn children of PPCM mothers treated with bromocriptine [22]. The study revealed that the infants of mothers in both treatment groups displayed normal weight-for-age, height-for-age, and survival at six months with no significant differences in their children's growth curves and no significant harm to their children [22]. The average growth in the children observed is an essential finding due to previous studies reporting the adverse impact of bromocriptine-induced ablactation on the health of the newborn children of bromocriptine-treated mothers who cannot breastfeed [41].

Limitations

This study is limited because we acquired results from single-center studies with small study populations and a short follow-up course. Notably, there is significant heterogeneity in the results reported between the studies due to differences in the bromocriptine regimen administered (2.5 mg daily versus 5 mg twice daily), study designs, inclusion criteria, definitions of acute PPCM, and the clinical outcomes measured. The reports on the clinical endpoints of children born to mothers diagnosed with PPCM are severely limited.

## Conclusions

Peripartum cardiomyopathy (PPCM) is a severe condition associated with pregnancy and the period after childbirth globally. When added to conventional heart failure (HF) treatment, bromocriptine enhances left ventricular function and decreases cardiovascular death in women presenting with this condition. This drug is primarily effective in patients presenting with symptoms before delivery and within the first month postpartum. No significant adverse events, including no events of thromboembolism, are associated with its use in acute PPCM. Given these results, bromocriptine should be considered a disease-specific treatment for PPCM, combined with regular HF treatment. However, large-scale multicenter studies with more extended follow-up periods are warranted to assess this more robustly. Of note, blinding study participants remains challenging because bromocriptine suppresses lactation, and therefore, women receiving the drug would not be able to nurse their infants while women on standard HF therapy would continue to breastfeed their infants. More studies are needed to report the clinical outcomes of newborn children of mothers who cannot breastfeed due to bromocriptine-induced ablactation. Finally, the optimal management of PPCM necessitates further research into alternative disease-specific treatments that target other molecules involved in the pathophysiological pathways of this disease.

## References

[1] Pearson GD, Veille JC, Rahimtoola S (2000). Peripartum cardiomyopathy: National Heart, Lung, and Blood Institute and Office of Rare Diseases (National Institutes of Health) workshop recommendations and review. JAMA.

[2] Sliwa K, Hilfiker-Kleiner D, Petrie MC (2010). Current state of knowledge on aetiology, diagnosis, management, and therapy of peripartum cardiomyopathy: a position statement from the Heart Failure Association of the European Society of Cardiology Working Group on peripartum cardiomyopathy. Eur J Heart Fail.

[3] Sliwa K, Mebazaa A, Hilfiker-Kleiner D (2017). Clinical characteristics of patients from the worldwide registry on peripartum cardiomyopathy (PPCM): EURObservational Research Programme in conjunction with the Heart Failure Association of the European Society of Cardiology Study Group on PPCM. Eur J Heart Fail.

[4] Sliwa K, Böhm M (2014). Incidence and prevalence of pregnancy-related heart disease. Cardiovasc Res.

[5] Kolte D, Khera S, Aronow WS (2014). Temporal trends in incidence and outcomes of peripartum cardiomyopathy in the United States: a nationwide population-based study. J Am Heart Assoc.

[6] Lima FV, Yang J, Xu J, Stergiopoulos K (2017). National trends and in-hospital outcomes in pregnant women with heart disease in the United States. Am J Cardiol.

[7] Patten IS, Rana S, Shahul S (2012). Cardiac angiogenic imbalance leads to peripartum cardiomyopathy. Nature.

[8] Hilfiker-Kleiner D, Sliwa K (2014). Pathophysiology and epidemiology of peripartum cardiomyopathy. Nat Rev Cardiol.

[9] Sliwa K, Förster O, Libhaber E, Fett JD, Sundstrom JB, Hilfiker-Kleiner D, Ansari AA (2006). Peripartum cardiomyopathy: inflammatory markers as predictors of outcome in 100 prospectively studied patients. Eur Heart J.

[10] Halkein J, Tabruyn SP, Ricke-Hoch M (2013). MicroRNA-146a is a therapeutic target and biomarker for peripartum cardiomyopathy. J Clin Invest.

[11] Haghikia A, Kaya Z, Schwab J (2015). Evidence of autoantibodies against cardiac troponin I and sarcomeric myosin in peripartum cardiomyopathy. Basic Res Cardiol.

[12] Forster O, Hilfiker-Kleiner D, Ansari AA (2008). Reversal of IFN-γ, oxLDL and prolactin serum levels correlate with clinical improvement in patients with peripartum cardiomyopathy. Eur J Heart Fail.

[13] Hilfiker-Kleiner D, Kaminski K, Podewski E (2007). A cathepsin D-cleaved 16 kDa form of prolactin mediates postpartum cardiomyopathy. Cell.

[14] Saint Croix GR, Ibrahim M, Chaparro S (2017). Use of bromocriptine in the management of peripartum cardiomyopathy: a systematic review. Circ Cardiovasc Qual Outcomes.

[15] Bauersachs J, König T, van der Meer P (2019). Pathophysiology, diagnosis and management of peripartum cardiomyopathy: a position statement from the Heart Failure Association of the European Society of Cardiology Study Group on peripartum cardiomyopathy. Eur J Heart Fail.

[16] Ponikowski P, Voors AA, Anker SD (2016). 2016 ESC guidelines for the diagnosis and treatment of acute and chronic heart failure: the task force for the diagnosis and treatment of acute and chronic heart failure of the European Society of Cardiology (ESC) developed with the special contribution of the Heart Failure Association (HFA) of the ESC. Eur Heart J.

[17] Mebazaa A, Yilmaz MB, Levy P (2015). Recommendations on pre-hospital & early hospital management of acute heart failure: a consensus paper from the Heart Failure Association of the European Society of Cardiology, the European Society of Emergency Medicine and the Society of Academic Emergency Medicine. Eur J Heart Fail.

[18] Hilfiker-Kleiner D, Haghikia A, Berliner D (2017). Bromocriptine for the treatment of peripartum cardiomyopathy: a multicentre randomized study. Eur Heart J.

[19] Hilfiker-Kleiner D, Haghikia A, Masuko D (2017). Outcome of subsequent pregnancies in patients with a history of peripartum cardiomyopathy. Eur J Heart Fail.

[20] Haghikia A, Schwab J, Vogel-Claussen J (2019). Bromocriptine treatment in patients with peripartum cardiomyopathy and right ventricular dysfunction. Clin Res Cardiol.

[21] Page MJ, McKenzie JE, Bossuyt PM (2021). The PRISMA 2020 statement: an updated guideline for reporting systematic reviews. BMJ.

[22] Sliwa K, Blauwet L, Tibazarwa K (2010). Evaluation of bromocriptine in the treatment of acute severe peripartum cardiomyopathy: a proof-of-concept pilot study. Circulation.

[23] Yaméogo N, Kagambèga L, Seghda A (2017). Bromocriptine in management of peripartum cardiomyopathy: a randomized study on 96 women in Burkina Faso. J Cardiol Clin Res.

[24] Haghikia A, Podewski E, Libhaber E (2013). Phenotyping and outcome on contemporary management in a German cohort of patients with peripartum cardiomyopathy. Basic Res Cardiol.

[25] Carlin AJ, Alfirevic Z, Gyte GM (2010). Interventions for treating peripartum cardiomyopathy to improve outcomes for women and babies. [PREPRINT]. Cochrane Database Syst Rev.

[26] Villanueva DLE, Evangelista LK, Espanillo-Villanueva MC, Anonuevo JC (2020). Use of bromocriptine for the treatment of peripartum cardiomyopathy: a meta-analysis of randomized controlled trials. Int J Clin Cardiol.

[27] Negoro S, Kunisada K, Fujio Y (2001). Activation of signal transducer and activator of transcription 3 protects cardiomyocytes from hypoxia/reoxygenation-induced oxidative stress through the upregulation of manganese superoxide dismutase. Circulation.

[28] Bartoli M, Platt D, Lemtalsi T, Gu X, Brooks SE, Marrero MB, Caldwell RB (2003). VEGF differentially activates STAT3 in microvascular endothelial cells. FASEB J.

[29] Ware JS, Li J, Mazaika E (2016). Shared genetic predisposition in peripartum and dilated cardiomyopathies. N Engl J Med.

[30] Ersbøll AS, Damm P, Gustafsson F, Vejlstrup NG, Johansen M (2016). Peripartum cardiomyopathy: a systematic literature review. Acta Obstet Gynecol Scand.

[31] Bello N, Rendon IS, Arany Z (2013). The relationship between pre-eclampsia and peripartum cardiomyopathy: a systematic review and meta-analysis. J Am Coll Cardiol.

[32] Rajakumar A, Michael HM, Rajakumar PA (2005). Extra-placental expression of vascular endothelial growth factor receptor-1, (Flt-1) and soluble Flt-1 (sFlt-1), by peripheral blood mononuclear cells (PBMCs) in normotensive and preeclamptic pregnant women. Placenta.

[33] Bello NA, Arany Z (2015). Molecular mechanisms of peripartum cardiomyopathy: a vascular/hormonal hypothesis. Trends Cardiovasc Med.

[34] Ntusi NB, Mayosi BM (2009). Aetiology and risk factors of peripartum cardiomyopathy: a systematic review. Int J Cardiol.

[35] Bauersachs J, Arrigo M, Hilfiker-Kleiner D (2016). Current management of patients with severe acute peripartum cardiomyopathy: practical guidance from the Heart Failure Association of the European Society of Cardiology Study Group on peripartum cardiomyopathy. Eur J Heart Fail.

[36] Arrigo M, Blet A, Mebazaa A (2017). Bromocriptine for the treatment of peripartum cardiomyopathy: welcome on BOARD. Eur Heart J.

[37] Del Zotto E, Giossi A, Volonghi I, Costa P, Padovani A, Pezzini A (2011). Ischemic stroke during pregnancy and puerperium. Stroke Res Treat.

[38] Hopp L, Haider B, Iffy L (1996). Myocardial infarction postpartum in patients taking bromocriptine for the prevention of breast engorgement. Int J Cardiol.

[39] Loewe C, Dragovic LJ (1998). Acute coronary artery thrombosis in a postpartum woman receiving bromocriptine. Am J Forensic Med Pathol.

[40] Nagaki Y, Hayasaka S, Hiraki S, Yamada Y (1997). Central retinal vein occlusion in a woman receiving bromocriptine. Ophthalmologica.

[41] Fett JD (2008). Caution in the use of bromocriptine in peripartum cardiomyopathy. J Am Coll Cardiol.

